# Health care is the new battlefront for anti-DEI attacks

**DOI:** 10.1371/journal.pgph.0003131

**Published:** 2024-04-24

**Authors:** Oni J. Blackstock, Jessica E. Isom, Rupinder K. Legha

**Affiliations:** 1 Health Justice, New York, New York, United States of America; 2 Vision for Equity LLC, Boston, Massachusetts, United States of America; 3 Yale School of Medicine, New Haven, Connecticut, United States of America; 4 Antiracism in Mental Health Fellowship, Los Angeles, California, United States of America; Wayne State University School of Medicine, UNITED STATES

## Battlefront of bold backlashes

Health care is the new battlefront for anti-diversity, equity, and inclusion (DEI) attacks. It reflects a broadening of the wave of state-level anti-DEI in higher education legislation that has surged through the country with 65 anti-DEI bills introduced since 2023, eight of which have become law [1]. These anti-DEI efforts are also tightly linked to the 2022 US Supreme Court reversal of Roe v. Wade and the anti-trans and anti-abortion legislation that has swept through the country. Moreover, they are a part of a larger centuries-old pattern of racial progress and subsequent rageful backlash. The impact of growing attacks on DEI initiatives within healthcare portends potentially devastating consequences for existing efforts to diversify the healthcare workforce in service of an increasingly diverse nation and on existing racialized health inequities in the US.

## Challenging the legality of efforts to diversify the healthcare workforce

Anti-DEI efforts in health care include attacks on both private and public sector institutions, the latter of which have included lawsuits at the state and federal levels. At the state level, medical boards, which license, monitor, and discipline physicians, have been targeted. A suit against the Medical Board of California aims to halt law AB241 which requires one hour of training in implicit bias as continuing medical education for physicians. Studies have shown [2] that health care providers often have negative biases towards Black patients, as well as other racially and ethnically minoritized patients, and that these biases are correlated with poorer communication and lower quality of care, likely contributing to existing healthcare inequities. Lawsuits against the Tennessee and Louisiana medical boards are challenging the legality of policies ensuring representation of people from minoritized groups. Such requirements for board’s membership can help to ensure that their policymaking considers marginalized communities’ perspectives and reduce barriers to licensure for health professionals from these communities. At the federal level, Attorneys General from Alabama, Arkansas, Kentucky, Louisiana, Missouri, and Montana led by the State of Mississippi have waged a lawsuit against the U.S. Department of Health and Human Services, seeking to eliminate anti-racism plans as an eligible "improvement activity” for the Centers for Medicare & Medicaid Services’ Merit-based Incentive Payment System for healthcare providers; the anti-racism plans are intended to decrease health inequities. Most recently, North Carolina Representative Greg Murphy, MD, introduced a bill that would strip federal funding from medical schools with DEI programs and initiatives.

In the private sector, a lawsuit against a medical services company offering financial incentives to Black physicians to join was dismissed after the company said they would end the program. Already underrepresented in medicine, Black physicians often have the highest medical school debt burden of any racial/ethnic group [3], largely due to structural racism which has prevented Black families from accumulating wealth, for example, through home ownership. This has negative ramifications for being able to take advantage of cost-sensitive professional development opportunities such as professional medical organization membership and participation. Another court case targeted a health services research journal offering a health equity fellowship to specific minoritized groups underrepresented in the field. The journal removed that explicit requirement, and the case was dismissed. Notably, all of these cases were brought by the group, Do No Harm, which purports to want “to help protect patients and physicians from discriminatory and divisive ideologies”, but whose actions, including providing support to state legislative efforts against offering transgender affirming care, demonstrate otherwise.

## Efforts from within organized medicine to dismantle DEI initiatives

More recently, anti-DEI efforts within medical professional organizations have gained steam. A group of members of the American Academy of Dermatology (AAD) proposed a policy resolution, which was previously accessible online, to dismantle all the AAD’s DEI programs [4]. The resolution begins with acknowledging the positive role of DEI given the existing disparities within the specialty yet then veers into calling DEI “a political movement” which had led to “the control of speech and the stifling of diversity of thought”. Then, in what is now becoming an unfortunately common trope, the proposed resolution links DEI efforts to antisemitism. This is asserted despite the core values of DEI and antiracism being completely antithetical and in opposition to antisemitism. Fortunately, because of vigorous advocacy from Black and other members within the AAD, the resolution was rejected and a separate proposed resolution, which called for expansion of the DEI programs within AAD, accepted. Despite this positive outcome, it is likely, given national trends, that similar anti-DEI efforts will spread to other health professional organizations.

## Implications for U.S. healthcare workforce diversity and health equity

Given existing and widening racialized health inequities in the US, it is critically important, even lifesaving, that the healthcare workforce be reflective of this country’s population. Black, Latinx, and Indigenous physicians currently account for 5.0%, 5.8%, and 0.3% of physicians [5], respectively, despite accounting for 13.6%, 19.1%, and 1.3% of the general population [6]. Asian American doctors remain underrepresented in leadership positions within medicine [7]. In fact, the numbers of Indigenous people and Black men enrolling in medical school has declined over the past 40 years; the latter of which has been referred to as “an American crisis”. Racial concordance (when the provider and patient share the same race) is associated with numerous positive health outcomes for Black patients including being more likely to follow health care provider guidance [8] and to report receiving preventive care and needed care [9], and even a reduction in infant mortality [10]. In counties where there are more Black physicians Black people live longer [11]. Black, Latinx, and Asian American physicians are more likely to provide health care in communities most impacted by health inequities [12]. Combined with the Supreme Court’s decision to end affirmative action in higher education and the mounting exodus of physicians from the field, anti-DEI efforts may disproportionately drive out racially and ethnically minoritized physicians from the workforce, which will likely have literal life and death consequences for minoritized communities.

Medical training regulatory agencies, like the AAMC and ACGME, increasingly recognize the imperative for health professionals to competently care for marginalized communities and understand the role of social and structural determinants of health, including racism, in influencing health outcomes. Yet training standards and clinical guidelines mandating these practices still do not exist. Moreover, medical training and professionalization remain an abusive experience for many, and, in particular, for marginalized individuals [13], making the need for DEI initiatives for the next generation of health care providers even more crucial.

## A call to action to health care institutions and health professional organizations

As racially minoritized women physicians whose primary work is based in antiracism within medicine, our response to these anti-DEI attacks is to educate and organize. Health professionals and professional organizations must increase their awareness of anti-DEI misinformation and propaganda, and communicate clearly about the tremendous value of diversity, equity, inclusion, and antiracism for achieving health equity in this country. Already, health professionals are pushing back against recent anti-DEI attacks using tools such as social media and petitions.

Moreover, in this moment of increasing urgency, health care institutions and professional organizations must double down and recommit to their DEI and antiracism efforts drawing on best practices [14], rather than back down and lose whatever traction has been gained. In the past, medical schools and medical professional organizations have been on the wrong side of progress [15] impeding Black and other minoritized people’s advancement in the field of medicine. Today, these institutions and organizations have a tremendous opportunity to be on the right side of history by honoring and building upon their commitments to justice, equity, diversity, inclusion, belonging, and antiracism, which they announced in response to the events of 2020 as they acknowledged their own racist legacies. Change at any level demands that we ride the waves of resistance knowing that with any semblance of progress comes backlash. As such, we call on those within and aligned with the medical profession to embody in language and practice our profession’s core values of beneficence, non-maleficence, and justice. Now is not the time for healthcare institutions and professional organizations to retreat, but instead, to be ready and prepared to rebuff anti-DEI attacks by deepening their commitment to and investment in embedding the values of diversity, equity, inclusion, and antiracism within their organizations.
